# Transmission of Tuberculosis through Meat and Milk1Presented at the Thirty-eighth Annual Convention of the American Veterinary Medical Association, Atlantic City, N. J., September, 1901.

**Published:** 1901-12

**Authors:** John J. Repp

**Affiliations:** Ames, Iowa, Professor of Pathology and Therapeutics, and Veterinarian to the Experimental Station, Iowa State College


### TRANSMISSION OF TUBERCULOSIS THROUGH MEAT AND MILK.[1]

By John J. Repp, V.M.D.,
AMES, IOWA,
PROFESSOR OF PATHOLOGY AND THERAPEUTICS, AND VETERINARIAN TO THE EXPERIMENTAL STATION, IOWA STATE COLLEGE.

(Concluded from page 691.)

### II.—Transmission to Man.

### *A. By Meat.*

1. By Artificial Methods. There is no evidence of any sort on this point.

2. By Natural Methods. The evidence on this score is only presumptive. However, it is well known that since ancient times legislation and sanitary regulations have been moulded around the presumption that the meat of highly tuberculous animals is dangerous as human food on account of the risk of conveying the disease to man through this medium. At this time every civilized nation that has any legislation or sanitary regulations in regard to the meat of tuberculous animals provides that

[1] Presented at the Thirty-eighth Annual Convention of the American Veterinary Medical Association, Atlantic City, N. J., September, 1901.

such meat shall either be condemned or that it shall be sold under declaration. These laws and regulations are based upon the analogy between tuberculosis in animals and the same disease in man, and the fact of the intertransmissibility of this disease among the various species of animals. Whether this evidence warrants such restriction on the use of meat or not has not yet been positively demonstrated, and, on account of the impracticability of direct experiment with human beings, we will almost certainly never be able to make such demonstration. The question must be decided upon the evidence we already have, and upon the additional evidence of the same character which from time to time may be added.

Of course, cooking of meats as it is usually practised effectually disposes of most of such danger as would exist if meat were eaten raw and without any attempt at sterilization. On the other hand, there is not one whit of evidence that tuberculosis is not to some extent transmitted from animal to man through ingestion of meat. All the evidence we have indicates that such transmission occurs to a limited extent.

### *B. By Milk.*

1. By Artificial Methods. No evidence.

2. By Natural Methods. The evidence of transmission in this manner consists of recorded observations which have been made in case of human beings who have used the milk of tuberculous cows.

Olivier[46] reports that in a young ladies' boarding-school five girls, the children of healthy parents, died of tuberculosis of the intestines. The cow which had for years supplied the school with milk was found to have generalized tuberculosis, including the udder.

Two daughters of a Scotch family of good health, who were brought up on milk of tuberculous cows, died of tuberculosis. Two sons in the same family who did not use the milk remained healthy.[47]

Stang[48] reports the case of a five-year-old boy of sound parentage and ancestry who died of tuberculosis. The cow whose milk this boy used was found badly tuberculous.

Demme[49] reports the cases of four infants in the Children's Hospital at Berne, the offspring of sound parents, that died of intestinal and mesenteric tuberculosis. He was able to exclude all other sources of infection, and to decide that they had been infected by the ingestion of the milk of tuberculous cows.

Hills[50] mentions the case of a child twenty-one months old, of a friend of his, which drank the milk of a highly tuberculous cow for one week while on a visit to his uncle, and three months later this child died of intestinal tuberculosis. Other sources of infection could be excluded. A second child, brought up on sterilized milk, is still healthy.

Hills[51] also reports the death of a boy, aged four years, at Yonkers, N. Y., from tubercular meningitis. The infection was traced to the milk of two cows, of whose milk this boy had drunk, and which proved on autopsy to be tuberculous.

Ernst[52] reports the death of three children of one family from tuberculosis. These children had used the milk of a cow which later died of advanced tuberculosis, including the udder

Leonhardt[53] reports the death, from tuberculosis of the meninges, intestines, and mesentery, of two children fed on milk of a tuberculous cow.

Sontag[54] reports the case of a six-months-old child, of healthy parents, who died of tuberculosis, and who had been fed on the milk of a tuberculous cow.

Hermsdorf[55] has reported the case of a child, dead of intestinal tuberculosis, who had been fed on the milk of a tuberculous cow.

Rich[56] reports that a young man of healthy parents, who died of tuberculosis, had used plentifully of the milk of a herd of seventy-four cattle, sixty-five of which were tuberculous, some of them markedly so. Also, another young man who died of tuberculosis.

Two months later Rich destroyed eighty cattle out of the herd of the family—that is, about 90 per cent. of the entire herd. Also, a young woman who died of tuberculosis, and a month later the cow whose milk she had used died of advanced tuberculosis.

Thorne[57] reports that twenty-two physicians out of 339 practising in Ohio replied in the affirmative to the question, "Have you been able to trace any cases of tubercular disease to the milk of unhealthy cows?" and that thirty-three replied affirmatively to the question, "Have you had reason to suspect the origin of tubercular disease in older children or adults to be in the milk-supply or meat-supply?"

This series of experiments and observations has been selected from the literature with the greatest care. Any reports which appeared not to be well authenticated, or of a doubtful nature, have been excluded. Besides this mass of positive evidence there is much more that, while not so positive, is not less convincing. It appears that the evidence collected by Thorne from physicians in Ohio is especially valuable not only in itself, but because it indicates what might be learned by addressing the same series of questions to physicians throughout the world.

Still further corroborative evidence is offered in the fact that such great numbers of bottle-fed children die of abdominal tuberculosis, and, in fact, that tuberculosis of adults is decreasing in many places, while tuberculosis of infants and small children does not show this marked decrease and is, in fact, increasing. Death from tuberculosis in all its forms in England and Wales has decreased 39.1 per cent. in the past thirty-five years. The greater part of this diminution was in the lung forms of this disease; for the same period the intestinal form has decreased only 8.5 per cent. It is noteworthy that, in the same period, the increase in abdominal tuberculosis in children under one year has been 27.7 per cent. Northrup[58] and Still[59] have presented statistics of autopsy in children which show that the pulmonary form of the disease is most common—that is, that the pulmonary lesions are primary, thus refuting the conclusion of most other pathologists, also based on autopsy. Koch,[60] in his very recent (July 23, 1901) address before the British Congress on Tuberculosis, has made reference to the findings of several physicians, including himself, in support of a similar allegation. This difference of conclusion can hardly be accounted for except upon the supposition that it is due to a difference in judgment among pathologists as to what is a primary and what a secondary lesion. This is a point on which the pathologist must exercise great care. He must not in this connection overlook the fact that, even though the most marked lesions appear in the thoracic cavity, this does not exclude primary infection by way of the digestive tract, because, as is well known, the infective agent has easy channels of passage from the abdominal cavity into the thoracic cavity by way of the thoracic duct and the vena cava. Theobald Smith[61] calls attention to this migration of the disease as having been observed by him and others (Spengler) experimentally. Even assuming the conclusions of Northrup and Still to be correct, although they are contrary to the opinions generally held, there is still left a large percentage of cases to be accounted for by intestinal infection. In 269 tuberculous children on whom Still made autopsies he considered that the channel of infection was the intestine in 53 cases and the lung in 105 cases. This is enough to warrant the most earnest crusade against the source of such infection.

However, neither animal inoculation, with its unmistakable results, so far as the animals themselves are concerned, nor the recorded observations of almost doubtless transmission to man through the milk and meat prove with the positiveness of a demonstration that tuberculosis is transmissible to man through the milk and meat. Yet to nearly all students of this subject this is convincing proof, and we find the results of it in the protective legislation which everywhere abounds.

We have still further and more valuable evidence, which may now be taken up.

Whether or not tuberculosis of animals is transmissible to man depends upon whether or not the tubercle bacillus of the animal species is pathogenic for man. If this could be demonstrated we would have a positive proof that would be beyond all question. We know that the virulent tubercle bacillus of the bovine species exists in the meat and milk products of these animals in many cases where they are tuberculous, and we know that man consumes these products thus bearing the bacillus. Therefore, if the pathogenicity of this animal tubercle bacillus for the human species can be proved, the question is no longer debatable. The following examples are offered for consideration. In my opinion they are capable of supplying the evidence we need.

Tscherning,[62] of Copenhagen, attended a veterinarian who had cut his finger in making an autopsy on a tuberculous cow. The wound healed, but there remained a swelling which soon ulcerated and refused to heal, so that the whole tumefied mass had to be cut out. The microscope revealed the distinct tuberculous process and the presence of the characteristically staining bacilli.

Pfeiffer[63] attended, at Weimar, a veterinarian named Moses, aged thirty-four years, of good constitution, and without hereditary disposition, who, in 1885, cut his right thumb deeply in making an autopsy on a tuberculous cow. The wound healed, but six months later the cicatrix still remained swollen, and in the autumn of 1886 the man had pulmonary tuberculosis, with bacilli in his sputa, and death occurred in two and a half years after the wound. Autopsy revealed tuberculosis of the joint of the wounded thumb, and in the lungs extensive tubercles and vomicæ.

Law[64] reports that a young veterinary friend of his, who was inoculated in the hand in opening a tuberculous cow, suffered from a tumefaction of the resulting cicatrix, with tubercle bacilli.

Rich[65] reports that a man cut his finger on a spicule of bone while making a post-mortem examination of tuberculous cows, and that in a few weeks he developed a tuberculous joint, and a few months later showed unmistakable signs of phthisis.

Ravenel[66] reports the case of a veterinarian who cut the knuckle of his finger while making a post-mortem examination of a tuberculous cow. The wound healed badly, remained swollen, and showed decided tendency to ulcerate. Removal of the cicatricial mass was practised, and the tissues were sent to him for examination. They showed typical tubercular lesions, with giant-cell formation. I am well acquainted with this case myself, and believe it to be an undoubted case of direct transmission of tuberculosis from cow to man by inoculation. This veterinarian told me that he did not become alarmed about the wound on his finger until he noticed a swelling and tenderness of the lymphatic glands on the inside of the elbow.

Ravenel[67] also reports the following two cases:

"On January 1, 1900, my assistant, Mr. G., while performing a post-mortem on a goat which had succumbed to experimental inoculation with a culture of bovine tubercle bacillus, scratched his knuckle on the broken ends of the ribs. Within a half-hour the wound was washed out with a 1 to 1000 solution of mercuric chloride and sealed. It healed promptly, but about three weeks after became reddened, swollen, and sensitive, especially on motion. It was protected, but grew worse, and on February 27th Dr. C. H. Frazier excised the nodule, with a margin of healthy skin. With one-half of the nodule we inoculated two guinea-pigs subcutaneously and the other part was prepared for sections. These sections show an infiltration-process which encroaches on the papillary layer of the skin, some of the papillæ being destroyed. No typical giant cells can be made out. None of the sections was stained for tubercle bacilli. On May 5th one of the inoculated pigs died, and the other was killed. Both of them showed a generalized tuberculosis involving the chest cavity as well as the abdomen. There has been no return of the lesion so far."

"The other case is that of a well-known veterinarian of Philadelphia, who, in making an autopsy on a tuberculous cow, wounded the knuckle of his forefinger. Between three and four weeks after the scar was noticed to be enlarged, reddened, and somewhat sensitive. As it showed no tendency to improve, but rather grew worse, some six weeks after it was first noticed it was excised by Dr. H. W. Cattell and the wound cauterized with bromine, since which there has been no return. The nodule was examined by Dr. John Guitéras, who demonstrated its tuberculous nature by finding tubercle bacilli in sections."

Further evidence than this is not on record, so far as I have been able to ascertain. Whatever our conclusions may be, they must be drawn from this evidence, together with some corroborative evidence of another character which will be referred to later on. Those who are in search of more convincing evidence of the pathogenicity of the bacillus of animal tuberculosis for man must be informed that it is not to be had without direct experimental inoculation or experimental feeding of members of the human species with tuberculous products of animals. That this is not likely to be done it is needless to state. It is not impossible that someone may apply to tuberculosis experiments of the same nature as those described by Arning,[68] in which a criminal was inoculated with leprous material. Certainly this sort of experimentation might be done in case of criminals, but such experimental work can never be done in case of children. Hence the relation between the milk of tuberculous cows and tuberculosis in children will never be decided on any evidence so positive as this.

Adami[69] considers as doubtful the conclusion that there has been a causal relation between the milk used and the tuberculosis of the persons using it, or between the wounds received while making autopsies and the subsequent tuberculosis in cases such as those cited in this article. He bases his opinion on the fact of the impossibility of total exclusion of all other sources of infection. One who puts forth such a statement as an argument does not realize the abyss of fatalism into which he would plunge science in all attempts at discovering the source of infection in case of any of the infectious diseases; especially would this be the case in such a chronic disease as tuberculosis. If the argument were valid it would apply to all diseases. It is not possible to absolutely and totally exclude all other sources of infection in any case. To do this would demand on the part of the experimenter and observer a knowledge of all possible sources of infection, other than the one under consideration, that might be active in a given case; and, further, a knowledge of altogether hidden and entirely unknown causes which might act. Unless this were done the same objection might be raised, viz.: The other causes have not yet all been excluded; there may be some source of infection of which neither you nor I, nor anyone else, has any knowledge—*reductio ad absurdum!* All we can do, and all that it is necessary to do, is to exclude all known or probable sources. We must decide upon an anchorage somewhere which the light of scientific knowledge points out. If we have done this, and get positive results in a sufficient number of cases, we have a legitimate right to draw conclusions accordingly. In the cases referred to it is to be considered that we have practically excluded all other sources of infection. Science does not require that more be done.

Some work has been done to ascertain whether or not there is any difference of morphology, character of culture, or virulence between the tubercle bacillus derived from man and that derived from animals, with the hope of incidentally shedding some light on the question of the transmissibility of tuberculosis from animal to man. It was taught by the earlier investigators from Koch forward, with few exceptions, that the bacilli from these two sources were virtually similar. This belief was held and taught until the recent experiments of Smith,[70] Pearson,[71] and Dinwiddie[72] were made, and which show that the bovine tubercle bacillus is distinctly more virulent for the species of animals thus far experimented upon than is the human bacillus, with a few exceptions, in which no difference in virulence was seen. Theobald Smith is probably the only one who has made a comparative study of the bacilli from the bovine and the human source, in culture and under the microscope, of sufficient extent to be of any value. He has noticed some points of difference, but none that, so far as we know, have any bearing upon the question under consideration. It is true that if there is a marked difference between the bacilli from the two sources it may in time be found out that, by a careful study of the bacilli themselves, we shall be able to differentiate cases of human infection from cases of bovine infection in man by an examination of the respective bacilli. However this may be, such a stage has not yet been reached. It may be that the cultures of human bacilli which Smith studied were all made from cases of human infection, and that the bacilli were adapted to the human system for generations. If a study of human bacilli from a case of supposed bovine infection could be studied side by side with bovine bacilli, the differences which Smith found might not be present. This seems to be a fertile field for investigation. Koch[73] expresses (July 23, 1901) vaguely the possibility of differentiating between intestinal tuberculosis in man of animal and of human origin by inoculation of cattle. If of animal origin, the cattle, he leads us to infer, will be infected, and if of human origin, the cattle will not be infected. He does not, however, offer the proof necessary to remove this from the realm of pure speculation.

On the other hand, it is not unlikely that the bovine tubercle bacillus, by passing through the bodies of several persons, or even one, may be so modified as to present under study the characters which we ascribe to the human bacillus. Weight is added to this view by the experiments of Nocard,[74] in which he succeeded, by means of cultures *in vivo*, in transforming the human bacillus into one of the avian type; also by the experiments of Dubard and Kral,[75] in which it was shown that the tubercle bacillus derived from fish was at first pathogenic for cold-blooded animals only, and grew only at a low temperature, but that by passage through a series of guinea-pigs and rabbits it became virulent for these animals, and that by frequent transplantation it was induced to grow at incubator temperature and produce a culture just like the human bacillus, from which it was supposed to have been at first derived.

Neither the work of Smith, Pearson, nor Dinwiddie adds or pretends to add anything positive to our knowledge of the transmissibility of tuberculosis from animal to man through the meat and milk. All evidence we have from this work is that afforded by an interpretation of the results and the use of them as a basis for reasoning about what the result would be if the terms of the experiment should be reversed and human beings were inoculated with bovine bacilli. By such an examination of these results we find that they are rightfully interpreted as antagonistic to the idea that tuberculosis is not communicated from animal to man through the meat and milk. Smith's experiments have evidently, because of careless reading, been used by some writers as proof of a non-transmissibility from the animal to the human being. On the contrary, Smith's experiments do not indicate this, nor does Smith claim that they do. This author says, after announcing his experiments, without any reference to these experiments, " it seems to me that, accepting the clinical evidence on hand, bovine tuberculosis may be transmitted to children when the body is overpowered by large numbers of bacilli, as in udder tuberculosis, or where certain unknown favorable conditions exist." Smith concludes his article by saying,[76] " if in this brief summary I have presented nothing but problems to be solved and doubts to be entertained, I feel quite," etc., thus indicating that he has proved nothing, but that he has only pointed the way toward the proper field of investigation—something he has certainly done. Dinwiddie does not touch upon these points.

The work of Smith and Dinwiddie, already referred to, which points to the conclusion that the bovine tubercle bacilli are more virulent[77] for a number of species than the human tubercle bacilli, and equally virulent[78] for others, certainly warrants us, if we draw any conclusion at all with reference to man, that the bovine tubercle bacilli are more virulent for man also. The extreme susceptibility of man to tuberculosis, indicated by the large percentage of the human race suffering from the disease, would further point to the soundness of such a conclusion. Manifestly, the opposite conclusion would be eminently unfair. It has been intimated by Conn[79] that "if the human bacillus is only slightly pathogenic for cattle, it is at least likely that the bovine variety may not be very dangerous to man." It would be as reasonable to say that, inasmuch as the American soldiers, who at one time during the Philippine war were armed with Springfield rifles which were of short range, were not dangerous to the Filipinos, neither were the Filipinos, at that time armed with long-range Mauser rifles, dangerous to the American soldiers.

I consider that there is another point worthy of notice. It is this: In the comparisons of the virulence of bovine and human tubercle bacilli which have been made by Smith and Dinwiddie they have compared the bacilli of human *sputum* with the bacilli of bovine *tissues*.

I believe that we cannot make a fair comparison between tubercle bacilli from the human and the bovine source unless all the conditions on both sides are as nearly similar as possible. We cannot make a fair comparison between tubercle bacilli derived from human *sputum* and those derived from bovine *tissues*. Smith has found by comparative study of the bacilli from these two sources in cultures that those from human sputum are more saprophytic in growth than those from bovine tissues. It may be that the more saprophytic the bacillus is the less virulent it is, and that this will account for the fact that the bacillus from human sputum shows itself to be less virulent than that from bovine tissues. It appears that in order to carry on absolutely fair comparative researches it would be necessary to watch the cases on both sides for a considerable time, to make out whether they are acute or chronic, and then take the germs from the tissues in the same part of the body of each, and from tissues which have undergone similar degeneration on account of the disease. Reliable work can be done only with cultures, and not with tissues. The reasons for this are easily apparent. Until this is done it seems to me that but little can be done on this part of the subject which will warrant us in drawing conclusions. If experiments be made in the manner suggested it may be that the difference in virulence between human and bovine bacilli would not appear, and that they may be found to be of equal or nearly equal virulence.

Inasmuch as Koch's address before the British Congress on Tuberculosis,[80] on July 23, 1901, has received so much attention and has been the occasion for so many unwarranted dicta, especially upon the part of those who oppose the doctrine of transmission of tuberculosis from animal to man, it is incumbent upon me to give it special notice here. First, I would notice a certain statement of his which has been the subject of the most misinterpretation. Koch has made within the last two years numerous inoculations and feedings of cattle with human tubercle bacilli from sputum in which he has failed to infect them. At the same time cattle inoculated or fed in the same manner with bovine tubercle bacilli from the lungs became badly infected. He concludes: "Considering all these facts, I feel justified in maintaining that human tuberculosis differs from bovine, and cannot be transmitted to cattle. It seems to me very desirable, however, that these experiments should be repeated elsewhere, in order that all doubt as to the correctness of my assertion may be removed." Koch has nowhere stated that because human tuberculosis cannot be transmitted to cattle neither can cattle tuberculosis be transmitted to man. Some critics of Koch's paper have made it appear that Koch has given expression to such a line of reasoning, but a careful reading of the paper will show that he has not done so. He does not use this argument at all when he comes to answer the question, "Is man susceptible to bovine tuberculosis?" So far as the answer to this question is concerned he wisely leaves his cattle experiments out of consideration. Second, he makes the following answer to the question, "Is man susceptible to bovine tuberculosis?": "Though the important question whether man is susceptible to bovine tuberculosis at all is not yet absolutely decided, and will not admit of absolute decision to-day or to-morrow, one is nevertheless at liberty to say that, if such a susceptibility really exists, the infection of human beings is but a very rare occurrence. I should estimate the extent of the infection by the milk and flesh of tuberculous cattle, and the butter made from their milk, as hardly greater than that of hereditary transmission, and I therefore do not deem it advisable to take any measures against it." Let us see what brings him to this conclusion. It is the result of his own observation and the observation of several other physicians, to wit, that *primary* tuberculosis of the human intestine is a very rare disease. His reasoning may be stated in this way: If tuberculosis were set up in the human being by ingesting the meat and milk of tuberculosis cattle the primary lesion would be in the intestine; primary tuberculosis of the human intestine is very rare; therefore, infection of the human being with tuberculosis by ingestion of meat and milk of tuberculous cattle is very rare.

The soundness of his reasoning should be examined into. In the first place, as already pointed out at another place in this article, there is room for difference of opinion in regard to the prevalence of primary tuberculosis of the human intestine, and the burden of proof is certainly on the side of those who claim that primary intestinal tuberculosis is rare. I may ask on which side tuberculosis of the peritoneum, liver, spleen, and mesenteric lymphatic glands should be counted. It would appear most reasonable to suppose that in these manifestations of tuberculosis the offending bacilli entered by way of the digestive tube; also, Koch's own experiments, detailed in his paper before the British Congress on Tuberculosis, in which he produced "tuberculosis of the lymphatic glands of the neck, and in one case a few gray nodules in the lung" of a pig, by feeding human sputum, and "tuberculous infiltration of the greatly enlarged lymphatic glands of the neck and of the mesenteric glands, and also extensive tuberculosis of the lungs and the spleen" in pigs by feeding bovine bacilli, certainly contradict his hypothesis, namely, "that a case of tuberculosis has been caused by alimenta can be assumed with certainty only when the intestine suffers first—that is, when a so-called primary tuberculosis of the intestine is found." Pearson[82] refers to experiments of his in which "it has been shown in the most unmistakable way by many feeding experiments . . . that, contrary to the early belief, animals fed tuberculous materials may develop primary pulmonary tuberculosis, and, in some instances, fail to show lesions in any other organ."

Upon this foundation Koch's conclusion rests. I can only add that the facts do not warrant so sweeping a conclusion.

After all, we must face the homely facts that we have in cattle and in man a very prevalent disease which we properly call by the same name—tuberculosis—in both species, and which presents very much the same pathological picture in both; also, that we find in sections from the tissues of tuberculous subjects of both these species myriads of germs which have practically the same morphology and the same staining reaction; also, that these germs from these two sources are found to have in a general way the same cultural qualities. These familiar characteristics, although they do not answer to the critical demands of precise science, should certainly lead us to ask for the most convincing proof that tuberculosis of man and tuberculosis of animals are two different diseases before we adopt the belief that they are.

It may be stated *en passant* that students of this subject of transmissibility of tuberculosis by meat and milk, whether they be individuals or commissions appointed to study the matter, are almost unanimous in their opinion that there is danger of such transmission, and that this danger is great enough to call forth protective legislation and regulation against these sources of danger. Attention may be called to the fact that the British Congress on Tuberculosis, before which Koch read the paper to which reference is made above, passed a resolution, after his paper was read and debated, recommending that the present restrictive regulations thrown around the sale and use of products of tuberculous cattle should be continued without any relaxation.

What evidence has brought them to such conclusion? Practically that which has been presented in this paper.

SUMMARY. The evidence presented here is:

1. That tuberculosis may be transmitted to animals through their eating the meat of certain other animals which are tuberculous, or by their being inoculated with it.

2. That tuberculosis may be transmitted to animals through their ingestion of the milk of certain cows which are tuberculous, or by their being inoculated with it, both when the udder of the cow is diseased and when it is healthy.

3. That, therefore, the meat and milk of certain tuberculous animals contain living, virulent tubercle bacilli.

4. That the tubercle bacilli of cattle are pathogenic for man.

5. That, therefore, the meat and milk of certain tuberculous animals are capable of producing tuberculosis in human beings who use these products as food.

PRACTICAL CONCLUSIONS. 1. *In Regard to Meat.* The meat of all food animals, especially cattle, is unfit for food when the animal is highly tuberculous; but is safe for food when the animal is only slightly or moderately tuberculous, especially so if the meat is well cooked, provided the tubercular tissues are eliminated.

2. *In Regard to Milk.* (*a*) The milk of a cow with tuberculous udder is always dangerous for food unless it is well sterilized.

(*b*) The milk of tuberculous cows with healthy udders is sometimes dangerous for food unless well sterilized. We cannot tell except by experiment, which is impracticable as a routine matter, when such milk is dangerous and when it is not; hence the milk of tuberculous cows without disease of the udder should always be looked upon with suspicion, and either not be used or be used only after sterilization.

(*c*) Tuberculous cows may be kept for breeding purposes, provided they are isolated, even from their own offspring, and their products sterilized before use; or,

(*d*) They may be slaughtered for food under conditions imposed by the conclusion stated above in regard to meat.

General Conclusion. All legislation and regulation should favor the disposition of tuberculous animals as suggested above, so far as meat and milk are concerned.

### References.

1. Law. Bulletin 65, Cornell Experimental Station, p. 134.
2. Loc. cit.
3. Walley. A Practical Guide to Meat Inspection, 3d ed., London and New York, 1896, p. 153.
4. Loc. cit.
5. Ibid., p. 155.
6. Ibid., p. 152.
7. Ibid.
8. Ibid.
9. Quoted by Law in Bulletin 65, Cornell Experimental Station, p. 133.
10. Loc. cit.
11. Report of Board of Agriculture of Pennsylvania, 1899, p. 380.
12. Friedberger and Fröhner's Veterinary Pathology, translation by Hayes, vol. ii. p. 179.
13. Bulletin 65, Cornell Experimental Station, p. 110.
14. Bergey. Veterinary Magazine, vol. iii. p. 726.
15. Walley. A Practical Guide to Meat Inspection, 3d ed., p. 152.
16. Report of Massachusetts Society for Promotion of Agriculture, 1894.
17. Ravenel. Journal of Comparative Medicine, December, 1897.
18. Report of National Veterinary Congress, London, 1891, vol. ii. p. 195.
19. Law. Bulletin 65, Cornell Experimental Station, p. 135.
20. Journal of Comparative Medicine, December, 1897.
21. Report of Wisconsin Experimental Station, 1894.
22. Journal of Comparative Pathology and Therapeutics, June, 1897.
23. Bureau of Animal Industry, Bulletin No. 7.
24. Rabinowitch and Kempner. Zeitschr. f. Hyg. u. Infectionskrank., May 20, 1899.
25. Loc. cit.
26. Ibid.
27. Ibid.
28. Lancet, No. 3933, p. 75.
29. Ibid., September 25, 1899, p. 840.
30. Philadelphia Medical Journal, December 30, 1899.
31. Zeitschr. f. Hyg. u. Infectionskrank., May 26, 1899.
32. Hygienische Rundschau, vol. ix., No. 2.
33. Report of Pennsylvania Board of Agriculture, 1899, p. 375.
34. Ibid., p. 370.
35. Ugesskrift for Landmand, 1892.
36. Report of Pennsylvania Board of Agriculture, 1897, p. 486.
37. Cornell Experimental Station, Bulletin 65, p. 136.
38. Report of Massachusetts Society for Promotion of Agriculture, 1894.
39. Loc. cit.
40. Report of Pennsylvania Board of Agriculture, 1899, p. 371.
41. Ibid., 1897, p. 486.
42. Vermont Experimental Station, Bulletin 42, p. 51.
43. Wisconsin Experimental Station Report, 1894, p. 201.
44. Report of Pennsylvania Board of Agriculture, 1897, p. 487.

45. Friedberger and Fröhner's Pathology, translation by Hayes, vol. i. p. 200.
46. Ostertag. Handbuch der Fleischbeschau.
47. Hills and Rich. Bulletin 42, Vermont Experimental Station, p. 55.
48. Law. Bulletin 65, Cornell Experimental Station, p. 137.
49. Loc. cit.
50. Bulletin Vermont Experimental Station, No. 42, p. 55.
51. Loc. cit.
52. Report of Massachusetts Society for Promotion of Agriculture, p. 4.
53. Watson. Report of New Hampshire Board of Health, 1892.
54. Loc. cit.
55. Ibid.
56. Veterinary Magazine, vol. ii. p. 729.
57. Ohio Experimental Station, Bulletin No. 108, p. 348.
58. In a paper read before the Philadelphia Pediatric Society, 1897.
59. British Medical Journal, August 19, 1899.
60. American Medicine, vol. ii. p. 148.
61. Journal Experimental Medicine, vol. iii. pp. 507, 508, 509.
62. Law. Bulletin 65, Cornell Experimental Station, p. 138.
63. Loc. cit.
64. Ibid.
65. Veterinary Magazine, vol. ii. p. 729.
66. JOURNAL OF COMPARATIVE MEDICINE, December, 1897.
67. Philadelphia Medical Journal, July 21, 1900, p. 125.
68. Virchow's Archiv, vol. cxxxiv. p. 319.
69. Philadelphia Medical Journal, December 30, 1899.
70. Transactions of Association of American Physicians, 1896, xi., and Journal of Experimental Medicine, vol. iii. pp. 451 to 511.
71. Unpublished communication from Dr. Leonard Pearson, Philadelphia, Pa.
72. Bulletin Arkansas Experimental Station, No. 57, pp. 46–95.
73. American Medicine, vol. ii. p. 148.
74. Annales de l'Institut Pasteur, September, 1898.
75. Report of Pennsylvania Board of Agriculture, 1899, pp. 420–426.
76. Journal Experimental Medicine, vol. iii. p. 541.
77. Smith and Pearson.
78. Dinwiddie.
79. Storr's Experimental Station Report, 1898, p. 38.
80. American Medicine, vol. ii. pp. 145–150.
81. Philadelphia Medical Journal, vol. viii. p. 184.

